# Using Transcriptomics to Identify Differential Gene Expression in Response to Salinity among Australian *Phragmites australis* Clones

**DOI:** 10.3389/fpls.2016.00432

**Published:** 2016-04-13

**Authors:** Gareth D. Holmes, Nathan E. Hall, Anthony R. Gendall, Paul I. Boon, Elizabeth A. James

**Affiliations:** ^1^Royal Botanic Gardens Victoria, MelbourneVIC, Australia; ^2^La Trobe Institute for Molecular Science, La Trobe University, BundooraVIC, Australia; ^3^Department of Animal, Plant and Soil Sciences, AgriBio, La Trobe University, BundooraVIC, Australia; ^4^Institute for Sustainability and Innovation, Victoria University, Footscray ParkVIC, Australia

**Keywords:** clonality, common reed, differential gene expression, *Phragmites australis*, salinity, salt tolerance, transcriptomics

## Abstract

Common Reed (*Phragmites australis*) is a frequent component of inland and coastal wetlands in temperate zones worldwide. Ongoing environmental changes have resulted in the decline of this species in many areas and invasive expansion in others. In the Gippsland Lakes coastal waterway system in south-eastern Australia, increasing salinity is thought to have contributed to the loss of fringing *P. australis* reed beds leading to increased shoreline erosion. A major goal of restoration in this waterway is to address the effect of salinity by planting a genetically diverse range of salt-tolerant *P. australis* plants. This has prompted an interest in examining the variation in salinity tolerance among clones and the underlying basis of this variation. Transcriptomics is an approach for identifying variation in genes and their expression levels associated with the exposure of plants to environmental stressors. In this paper we present initial results of the first comparative culm transcriptome analysis of *P. australis* clones. After sampling plants from sites of varied surface water salinity across the Gippsland Lakes, replicates from three clones from highly saline sites (>18 g L^-1^ TDS) and three from low salinity sites (<6 g L^-1^) were grown in containers irrigated with either fresh (<0.1 g L^-1^) or saline water (16 g L^-1^). An RNA-Seq protocol was used to generate sequence data from culm tissues from the 12 samples allowing an analysis of differential gene expression. Among the key findings, we identified several genes uniquely up- or down-regulated in clones from highly saline sites when irrigated with saline water relative to clones from low salinity sites. These included the higher relative expression levels of genes associated with photosynthesis and lignan biosynthesis indicative of a greater ability of these clones to maintain growth under saline conditions. Combined with growth data from a parallel study, our data suggests local adaptation of certain clones to salinity and provides a basis for more detailed studies.

## Introduction

*Phragmites australis* (Cav.) Trin. ex Steud or “Common Reed” is a rhizomatous perennial grass found in fresh and saline wetland systems throughout temperate regions of the world. The species is genetically complex with a range of ploidy levels including 2n = 3x, 4x, 8x, 10x, and 12x (Clevering and Lissner, 1999; Lambertini et al., 2006). Several studies have reported high levels of genetic diversity within, and among, populations of this species (see Lambertini et al., 2008; Achenbach et al., 2012; and references therein) which may confer a high level of phenotypic plasticity in response to environmental variability (Hansen et al., 2007; Achenbach et al., 2012; Eller and Brix, 2012). In addition, *P. australis* can reproduce both sexually and asexually further contributing to its success in establishing and persisting under a range of environmental conditions. However, its local abundance is affected by several environmental factors including variable levels of nutrients, water and salinity, niche availability and its genetic make-up (Eller et al., 2014). For example, Achenbach et al. (2013) found substantial differences in salt tolerance between clones of *P. australis* supporting the idea that varied clonal responses have a genetic basis.

The rapid expansion of *P. australis* across North America in recent decades has been due almost exclusively to a Eurasian lineage introduced in the late 19th century (Haplotype ‘M’ *sensu* Saltonstall, 2002). Salinity tolerance (coupled with clonal reproduction) is thought to underpin the invasiveness of this genetic lineage (Vasquez et al., 2005, 2006). DNA sequence variants and varied expression levels of genes associated with salinity tolerance have been observed among *Phragmites* lineages (Zhao et al., 2004; Takahashi et al., 2007a,b, 2009) and while salinity tolerance has been reported more generally for *P. australis*, study results vary in the effect of salinity levels on growth response (e.g., Pagter et al., 2009; Gorai et al., 2011; Yang et al., 2014).

The Gippsland Lakes in south-eastern Australia is an extensive Ramsar-listed wetland system of >60,000 ha, that experienced chronic salinization following the construction of a permanent channel to the sea in the late 19th century to improve boat access (Boon et al., 2015a). The effects were exacerbated in the mid-late 20th century when fresh water from inflowing rivers was increasingly regulated and extracted. This process has led to substantial ecological impacts on the Lakes’ ecosystem including a marked decline in the area of fringing *P. australis* beds (Bird, 1961, 1966; Boon et al., 2008, 2015a; Sinclair and Boon, 2012). However, an extensive field survey undertaken in 2014 suggested that *P. australis* has re-appeared in parts of the system from which it seems to have been formerly precluded (Bird, 1961; Boon et al., 2015b). This raises the question as to why reed beds can now grow in these areas and whether there has been an adaptive response to increased salinity.

Shoreline erosion and retreat is a serious problem for the Gippsland Lakes (Hart, 1921; Bird, 1966, 1983; Bird and Rosengren, 1974; Sjerp et al., 2002). Shoreline degradation is expected to become even more pronounced with projected rises in eustatic sea levels coupled with an increased frequency and severity of storm surges, both linked to climate change. *Phragmites australis* is arguably the plant species most capable of protecting shorelines in the Gippsland Lakes from these processes (Boon et al., 2015b,c). Accordingly there is currently great interest in rehabilitating shoreline vegetation, often focussing on the restoration of *Phragmites* beds. However, the species has displayed mixed responses to increasing salinity making it difficult to pinpoint the causes and to identify the best germplasm for restoration. This has prompted an interest in the rapid identification of salt tolerant lineages of *P. australis* that could be used in the rehabilitation of wetlands and lake foreshores in the Gippsland Lakes, but also more generally across coastal wetland systems. The Gippsland Lakes provide an ideal opportunity to compare the responses of clones to salinity depending on the salinity of source populations.

One approach to help understand the mechanisms involved in the response of organisms to stressors is to compare genes that are up- or down-regulated under controlled conditions. Identifying the gene transcripts in *P. australis* that correlate with salinity tolerance will provide a basis for understanding this environmentally important trait.

In this study, we utilize next-generation sequencing technology to identify culm-expressed genes in *P. australis* associated with exposure to saline water. Six clones were obtained from areas of low or high salinity across the Gippsland Lakes and grown in pot trials. Paired-samples of the clones from each site were irrigated with either fresh-, or highly saline water. Using an RNA-Seq approach, we sequenced transcriptomes from the culms of each of these twelve samples and identified genes differentially expressed among treatments.

We predicted that plants from low salinity sites when irrigated with highly saline water would display higher levels of up-regulation of stress response genes than would plants from high salinity sites. Plants from high salinity sites irrigated with fresh water should display the opposite response. We also hypothesized that because of local adaptation, plants from high salinity sites grown in saline water would display minimal changes to gene expression states compared with plants from low salinity sites grown in fresh water. In summary, in this paper we address two main questions: (1) which genes are expressed in the culms of *P. australis* in response to growth in highly saline water? (2) which genes are differentially expressed in the culms of plants sourced from low salinity sites compared to plants from high salinity sites when grown in saline water?

## Materials and Methods

### Growth Conditions

**FIGURE 1:**
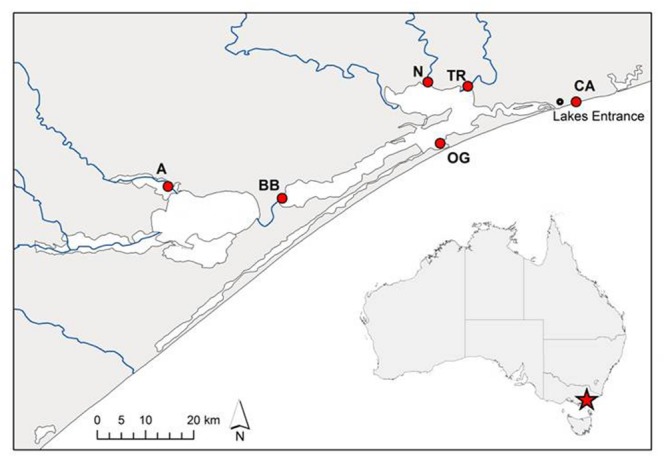
**Source sites of *Phragmites australis* clones from the Gippsland Lakes, south-eastern Australia used in this study.** Population codes are as listed in **Table 1**. The artificial opening of the lake system to Bass Strait is immediately south-west of the Lakes Entrance township while blue lines indicate inflowing rivers.

Sections of rhizome from six *P. australis* clones were harvested in September 2014 from several sites across the Gippsland Lakes area of south-eastern Australia (**Figure 1**; **Table 1**). These sites are described in Boon et al. (2015c). Rhizome sections were stored at 4°C in damp hessian bags until potted out. The surface-water salinities of the sites (**Table 1**) were determined using the total dissolved solids (TDS) function of a TPS WP-81 water-quality meter with a *k* = 10 temperature-compensating conductivity sensor (TPS Instruments, Brisbane, QLD, Australia). Although once-off ‘spot’ measurements, the surface-water salinities measured at each site agreed closely with long-term spatial patterns of water salinity across the Gippsland Lakes reported by Webster et al. (2001). To check whether *P. australis* plants could persist in apparently saline environments by accessing shallow lenses of fresh ground water, we also measured interstitial water salinity at various depths in the sediment at each site. These data will be reported in a separate paper. In summary, there was a good correlation (*r*^2^ = 0.41, *n* = 37) between salinity in surface water and interstitial water across the sites, strongly suggesting that the plants from saline sites were not subsidized by ground water lower in salinity than the overlaying water column (Boon et al., 2015c). Rhizomes of each clone were divided into short sections (typically 5-10 cm) and planted into 150 mm diameter plastic pots containing commercial potting medium with no added nutrients (Richgro All-purpose Potting mix, Richgro Garden Products, Jandakot, WA, Australia). Each pot was placed in a 9 L plastic bucket and positioned randomly in an outdoor trial plot at Victoria University, Werribee (Melbourne) in mid-October 2014. Six replicates of each of the six clones were prepared for each salinity treatment. During a preliminary four-week plant establishment phase, pots were kept wet by maintaining 1–2 cm of mains-sourced fresh water (<0.1 g L^-1^ TDS) in each bucket. This initial low water level was required to facilitate the development of the young plants (Doug Frood, pers. comm.). After the establishment period, water levels were increased to the height of the potting medium (i.e., the plants were inundated but not fully flooded). Nine weeks after planting, the water in each pot was replaced with mains water containing a dissolved salt mix of Oceanpure synthetic sea salt OP-50 (Commodity Axis, Camarillo, CA, USA) in a progression of 0, 2, 4, 8, or 16 g L^-1^. The highest salt concentration applied was approximately half of that found in typical sea water. At the same time, 5 g of slow-release fertilizer (Osmocote all-purpose, Scotts Australia, Bella Vista, NSW, Australia; 13.4% N, 1.0% P, 5.2% K, 7.5% S, 2.2% Ca, 0.3% Mg, 1.7% Fe, plus trace elements) was added to each pot. Water levels within each bucket were maintained by topping up with fresh water every two to three days and the water completely replaced with fresh solutions of the appropriate salinity every two weeks.

**Table 1:** Details of *Phragmites australis* source population within the Gippsland Lakes including surface water salinities measured as g L^-1^ total dissolved solids (TDS).

Population	Code	Water salinity (g L^-1^ TDS)
**Low salinity (‘L’)**		
Avon River	A	1.2
Tambo River	TR	3.1
Nicholson River	N	5.7
**High salinity (‘H’)**		
Bandon Bay	BB	18.6
Ocean Grange	OG	23.7
Cunninghame Arm	CA	31.2

*Populations are grouped by salinity level classes (low = < 6 g L^-1^; high = > 18 g L^-1^).*

### Sampling for Transcriptomics

After 8 weeks of exposure to salinity regimes, tissue samples from six clone pairs (one sample from the fresh; <0.1 g L^-1^ and one from the highly saline; 16 g L^-1^, irrigation treatments) were harvested on the same day between 11:15 and 13:00. No technical replicates were included. For each plant, a 2 cm-long culm section was sampled from within the leaf sheath directly below the topmost ligule from each of four actively growing stems. The tissue type and sampling position were chosen to obtain material at a comparable stage of development and to minimize external contaminants. The material was immediately immersed in 2 ml of chilled Qiagen RNAlater (Ambion Inc, Austin, TX, USA) and stored in darkness at 4°C until processed. Rhizome-tip samples were also harvested from one clone pair (TR) to aid in the assembly of a more complete reference transcriptome (see below) but are not reported.

### RNA Isolation

For each plant, an equal amount of tissue from each of the four sampled culms was pooled to give a total of 60–70 mg. This tissue was ground to a powder under liquid nitrogen using a mortar and pestle. While the sample was frozen, RLY buffer from an Isolate II RNA Plant Kit (Bioline, London, UK) was added and the material was allowed to thaw to a slurry before additional grinding was performed. Total RNA was subsequently isolated following the manufacturer’s protocol which included an on-column DNA digestion using DNase I. The RNA was eluted with 60 μl RNase-free water and the flow-through reapplied to the spin column for a second round of elution. Quality and quantity was assessed by electrophoresis on a 1.5% agarose gel and spectrophotometry using a NanoDrop 1000 v 3.7 (ThermoFisher Scientific, Wilmington, DE, USA). RNA was also isolated as above for small sections of the rhizome samples.

### cDNA Library Preparation and Sequencing

For each of the 14 samples (12 culms, 2 rhizomes), a polyA cDNA library was prepared from 4 μg of total RNA using a TruSeq Stranded mRNA LT Kit (Illumina, San Diego, CA, USA) following the manufacturer’s low sample (LS) protocol. A chemical fragmentation step of 30 s at 94°C as described in the Illumina protocol was used to prepare insert lengths between 130 and 340 bp with an aim of producing a final library size of c.450 bp. Fourteen complimentary adapters (Illumina) were chosen with the aid of a barcode diversity calculator^1^ and ligated to the sample inserts. For each of the 14 cDNA libraries, the fragment size average and range was assessed using a Bioanalyzer and associated DNA1000 reagent kit (Agilent Technologies, Santa Clara, USA) and the concentration determined using a Qubit 1.0 fluorometer (ThermoFisher Scientific, Wilmington, USA).

The concentration of each cDNA library was normalized to 10 nM before being pooled for processing. Paired-end sequencing of the libraries was undertaken at La Trobe University (Melbourne, VIC, Australia) on a HiSeq^TM^1500 platform after preparation with a TruSeq PE Cluster Kit v3-cBot-HS and a TruSeq SBS v3 kit (Illumina, San Diego, USA). The libraries were run across a proportion (c.74%) of two lanes on a flow cell.

### Transcriptome Assembly and Data Analysis

FastQ files from the sequencing run were de-multiplexed using CASAVA 1.8.2 software (Illumina). Read quality as determined by ‘phred’ scores was assessed using FastQC v0.11.4 (Andrews, 2010) on the LIMS High Performance Computing cluster (La Trobe University, Bundoora, Australia). High quality reads for all treatments, including the rhizome reads, were *de novo* assembled using Trinity version r20140717 (Grabherr et al., 2011) with stranded data and a minimum k-mer coverage of one to produce a reference transcriptome. Reads from individual samples were then mapped back to the reference using Bowtie (Langmead et al., 2009). The number of reads per gene model (hereafter referred to as a gene) was determined using RSEM (Li and Dewey, 2011) before annotation performed in Trinotate^2^. BLAST searches included Blastp and Blastx against the SwissProt database and Blastx nr and Blastn nt against the GenBank database.

Analysis of differential gene expression among treatments for culm samples was undertaken using *edgeR* v3.10.5 (Robinson et al., 2010) as implemented in Degust v0.2 with the False Discovery Rate (FDR) cut-off initially set to 1.0 and then to 0.05 for subsequent analyses. Using the latter FDR value, differences in expression levels of genes common to different treatments were assessed for samples grouped by source site salinity class (**Table 1**) and by irrigation treatment. Throughout the Results section, ‘L’ refers to plants sourced from low salinity sites and ‘H’ refers to plants sourced from high salinity sites as per **Table 1**. Salinity treatments were coded as ‘0’ for freshwater and ‘16’ for 16 g L^-1^ TDS. Gene expression levels from plants sourced from low salinity sites and irrigated with fresh water (L-0) were used as a baseline for comparison. Output from Degust was used to construct four-way Venn diagrams using Venny v.2.0.2 (Oliveros, 2015) to highlight differentially expressed genes common (or unique) to comparisons between specific pair-wise interactions of different treatments. The comparisons presented below highlight informative differences between plants sourced from ‘L’ and ‘H’ salinity sites and their response to irrigation with ‘0’ and ‘16’ treatments.

## Results

### RNA Isolation, Sequencing, and *De Novo* Assembly

Total RNA quality and yield was generally high with little degradation observed after electrophoresis and 260/280 nm absorbance ratios ranging between 2.11 and 2.19. Yields ranged between 10 and 41 μg from 60 to 70 mg of tissue. Sequencing of 12 pooled culm cDNA libraries resulted in approximately 28.6 Gb of data from 286 million 100-bp indexed reads (ave. 23.8 million reads/sample). Over 90% of the reads had phred scores ≥Q30 and 20% were ≥Q40. Reads from the two rhizome samples were used to improve the quality of subsequent assembly but are not reported further. *De novo* assembly of the reads from culm libraries resulted in the construction of 130,521 contigs across all samples ranging between 201 and 13,436 bp (ave. 732.1 bp). Included among the contigs with the highest raw read counts (c46264_g1 and c43507_g1) were sequences identified as the 18S ribosomal subunit and the 26S ribosomal subunit, respectively, indicative of incomplete non-polyA RNA exclusion during library preparation. Other sequences highly expressed across all samples include those identified as putatively coding for horcolin, phenylalanine ammonia-lyase, YLS9, dehydrin COR410, catalase isozyme 3, probable polyamine oxidase 2, mitochondrial glycine dehydrogenase (decarboxylating), chloroplastic cystathionine gamma-synthase 1, ethylene-responsive transcription factor RAP2-13-like, heavy metal-associated isoprenylated plant protein 26, NAC domain-containing protein 2, chloroplastic photosynthetic NDH subunit of lumenal location 5, cytochrome c, uncharacterized protein ycf68, chloroplastic glutamine synthetase, protein translation factor SUI1 homolog, metallothionein-like protein 4A, and rhodopsin.

### Differential Expression Analysis

**FIGURE 2:**
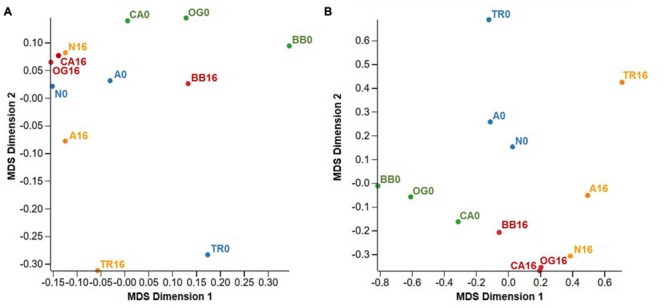
**Multi-dimensional scaling (MDS) plots summarizing gene expression profiles from *Phragmites australis* clones shared across two conditions (fresh and 16 g L^-1^ TDS salt water).** Blue labels: low salinity source site/fresh water irrigation (L-0); orange labels: low salinity source site/salt water irrigation (L-16); green labels: high salinity source site/fresh water irrigation (H-0); red labels: high salinity source site/salt water irrigation (H-16). **(A)** Represents all genes *n* = 62526, FDR cut-off = 1.0. **(B)** Includes only differentially expressed genes across treatments *n* = 1832, FDR cut-off = 0.5. Population codes are as per **Table 1**.

In total, 62,526 expressed ‘genes’ were shared between two or more treatment regimes when the FDR cut-off was set to 1.0 (i.e., all genes regardless of their differential expression level significance). The similarity between this suite of genes is represented in multi-dimensional space in **Figure 2A**. The MDS, very similar to a PCA plot, shows groupings of clones and condition when looking at all genes. When data from all clones treated with fresh water were combined and compared to those treated with saline water (L-0 + H-0 cf. L-16 + H-16) at an FDR cut-off of 0.05, 705 (1.13%) genes displayed significant differential expression levels. Of these, 349 were up-regulated in the ‘L-16 + H-16’ group and 356 were down-regulated (see **Supplementary Data Sheet 1**).

**FIGURE 3:**
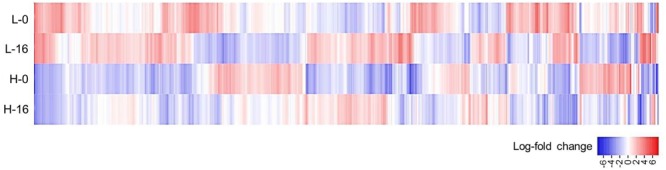
**Heat-map of *Phragmites australis* transcript data for plants sourced from low salinity sites (L) versus high salinity sites (H) and irrigated with fresh (0), versus 16 g L^-1^ TDS salt water (16).** The figure displays log_2_-fold change in average expression of 1832 gene models (horizontal axis) showing significant differential expression across the four data sets (FDR cut-off = 0.05). Genes up-regulated from the average are shown in red and genes down-regulated are shown in blue.

**FIGURE 4:**
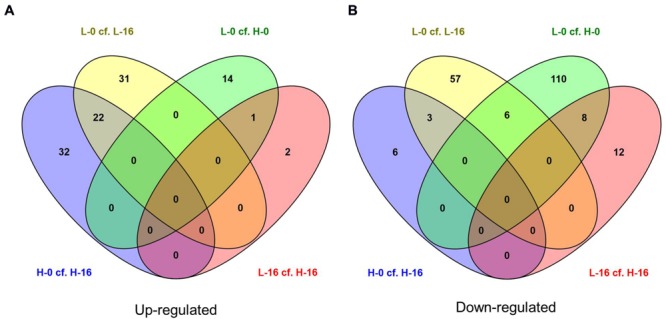
**Venn diagrams showing the number of **(A)** up-regulated and **(B)** down-regulated genes (FDR cut-off = 0.05) in salinity treatment comparisons for *Phragmites australis* clones from sites with differing water salinity levels (L = low salinity sites, H = high salinity sites; 0 = freshwater irrigation, 16 = 16g L^-1^ salt water irrigation).** Figures in the overlapping sections indicate the number of differentially expressed genes common to multiple pair-wise treatment regime comparisons. Lists of corresponding genes are presented in **Supplementary Data Sheet 2**.

When data from each individual treatment regime were compared (L-0 cf. H-0 cf. L-16 cf. H-16) at an FDR of 0.05, 1832 (2.93%) genes displayed significant differential expression levels in one or more of the regimes (**Figures 2B** and **3**). The clustering of clones sourced from Nicholson River (N) with Cunninghame Arm (CA) and Bandin Bay (BB) in **Figures 2A,B** was unexpected given the large differences in salinity at their source sites (**Table 1**). While the number of genes shared in the ‘L-0 cf. L-16’ comparison that showed significant up- and down-regulation were similar (53 and 60, respectively), the number of up-regulated genes shared in ‘H-0 cf. H-16’ was much greater than those down-regulated (54 and 9, respectively; **Figure 4**). In contrast, *P. australis* clones in the ‘L-0 cf. H-0’ comparison displayed a much lower number of shared up-regulated genes than those down-regulated (15 and 118, respectively).

There were no individual genes present in all four main pair-wise treatment regime comparisons (L-0 cf. L-16, H-0 cf. H-16, L-0 cf. H-0, and L-16 cf. H-16) that showed significant up- or down-regulation as indicated in the central overlapping segments in **Figures 4A,B**. The only treatment comparisons that showed up-regulated genes in common (**Figure 4A**) were ‘L-0 cf. L-16’ and ‘H-0 cf. H-16’ (22 genes) and ‘L-0 cf. H-0’ and ‘L-16 cf. H-16’ (one gene). Treatment comparisons that showed down-regulated genes in common (**Figure 4B**) were: ‘L-0 cf. L-16’ and ‘H-0 cf. H-16’ (3 genes); ‘L-0 cf. H-0’ and ‘L-16 cf. H-16’ (eight genes); ‘L-0 cf. L-16’ and ‘L-0 cf. H-0’ (six genes).

Differentially expressed genes in culm tissues representative of a general response to saline conditions independent of genotype are likely to include those common to the ‘L-0 cf. L-16’ and ‘H-0 cf. H-16’ comparisons. Of the 22 significantly up-regulated genes, notable Blast hits included six genes for which the products are chloroplastic (outer envelope pore protein 16-2; ATP-dependent zinc metalloprotease; glutamate synthase 2 [NADH]; ATP-dependent zinc metalloprotease; glucose-1-phosphate adenylyltransferase large subunit 1; 1,4-alpha-glucan-branching enzyme), Heat stress transcription factor C-2a, NADP-dependent malic enzyme, arginine decarboxylase 2, EID1-like F-box protein 3 and metal transporter Nramp5 (see **Supplementary Data Sheet 2** for further details). The three down-regulated genes found in both ‘L-0 cf. L-16’ and ‘H-0 cf. H-16’ comparisons were identified as similar to anthocyanidin 3-O-glucosyltransferase 2-like mRNA (*Setaria italica*), proline-rich protein (PRP) gene (*Saccharum* hybrid) and an unannotated sequence from *Oryza sativa* subsp. *japonica* chromosome 7. We also compared genes showing significant expression level differences in the combined (‘L-0 + H-0’) cf. (‘L-16 + H-16’) pair-wise data comparison which is likely to highlight the general response of *P. australis* to salinity (**Supplementary Data Sheet 1**).

Reponses to high salinity that are most likely to be associated with genotypic differences between *P*. *australis* clones are those unique to the ‘L-16 cf. H-16’ comparison. A total of 23 genes showed significantly different expression levels for this comparison and, of these, 14 were found only in this pair-wise comparison (**Table 2**). Of these, the two up-regulated genes returned Blast hits to the genes DIR1 (coding for dirigent protein 1) and CB48 (chlorophyll a/b binding protein 48). The 12 down-regulated genes included PER45 (Peroxidase 45), PCO1 (Plant cysteine oxidase 1), LAC15 (Laccase 15), PECT1 (ethanolamine-phosphate cytidylyltransferase), NO93 (early nodulin-93-like), and ankyrin repeat domain-containing protein 65-like.

**Table 2:** Differently expressed genes (FDR cut-off = 0.05) identified in *Phragmites australis* clones sourced from high salinity sites compared to low salinity sites when irrigated with highly saline water (16 g L^-1^ TDS).

Contig sequence ID	UniProt annotation	FDR	Log_2_-fold expression change	Gene product and organism	GO terms
**Up-regulated**					
c29327_g1^∗^	DIR1^1^	0.017	4.59	Dirigent protein 1 (*Arabidopsis thaliana*)	Cellular component; apoplast
c12017_g1^∗^	CB48^1^	0.021	3.94	Chlorophyll a–b binding protein 48, chloroplastic (*Zea mays*)	Biological process: photosynthesis, light harvesting; protein–chromophore linkage Molecular function: chlorophyll binding; metal ion binding Cellular component: chloroplast thylakoid membrane; integral component of membrane; photosystem I; photosystem II
c54138_g2	–	0.019	2.80	Hypothetical protein, mRNA (*Sorghum bicolor*)^4^	(Similar to Putative stress resistance-related protein)
**Down-regulated**					
c43433_g1	NUD14^1^	0.047	–2.67	Nudix hydrolase 14, chloroplastic (*Arabidopsis thaliana*)	Molecular function: ADP-glucose pyrophosphohydrolase activity; ADP-ribose pyrophosphohydrolase activity; ADP-sugar diphosphatase activity; metal ion binding Cellular component: chloroplast; chloroplast stroma
c45711_g1^∗^	–	0.030	–2.68	Uncharacterized protein, predicted (*Setaria italica*)^4^	–
c41377_g1^∗^	PER45^2^	0.019	–2.83	Peroxidase 45 (*Arabidopsis thaliana*)	Biological process; hydrogen peroxide catabolic process; response to oxidative stress Molecular function: metal ion binding; peroxidase activity Cellular component: extracellular region
c26253_g1^∗^	–	0.012	–3.16	ATP-dependent 6-phosphofructokinase 6-like mRNA – predicted (*Setaria italic*)^4^	–
c51225_g3	DIV^1^	0.045	–3.68	Transcription factor DIVARICATA (*Antirrhinum majus*)	Biological process: DNA binding Molecular function: determination of dorsal/ventral asymmetry; flower development; regulation of transcription, DNA-templated; transcription, DNA-templated Cellular component: nucleus
c56784_g6^∗^	–	0.002	–4.45	Phosphoethanolamine cytidylyltransferase (*Hordeum vulgare*)^3^	**–**
c55866_g4	RNHX1^1^	0.004	–4.53	Putative ribonuclease H protein (*Arabidopsis thaliana*)	Molecular function; metal ion binding; nucleic acid binding; RNA–DNA hybrid ribonuclease activity
c46015_g2	GSTX3^1^	0.019	–4.91	Probable glutathione *S*-transferase	Biological process: auxin-activated signaling pathway Molecular function: glutathione transferase activity
c61575_g1	–	0.019	–5.18	Predicted protein mRNA (*Hordeum vulgare*)^4^	–
c57868_g1	PLP3^2^	0.009	–5.81	Patatin-like protein 3 (*Oryza sativa* subsp. *japonica*)	Biological process: defense response; lipid catabolic process Molecular process: hydrolase activity
c26482_g3^∗^	–	0.049	–5.91	No significant hits	–
c71221_g1^∗^	–	0.004	–6.15	No significant hits	–
c29596_g1^∗^	PCO1^1^	0.047	–6.20	Plant cysteine oxidase 1 (*Arabidopsis thaliana*)	Biological process: anaerobic respiration; detection of hypoxia; peptidyl-cysteine oxidation; response to hypoxia Molecular function: cysteine dioxygenase activity; metal ion binding Cellular process: cytosol; nucleus
c48421_g1^∗^	NO93^1^	0.029	–6.38	Early nodulin-93 (*Glycine max*)	Biological process: Nodulation
c14700_g1^∗^	–	0.004	–6.50	Ankyrin repeat domain-containing protein 65-like mRNA – predicted (*Setaria italica*)^4^	–
c35884_g1^∗^	–	0.047	–7.10	Hypothetical protein, mRNA (*Sorghum bicolor*)^4^	–
c56558_g7	IMK2^1^	7.83e–^5^	–7.39	Probably inactive leucine-rich repeat receptor-like protein kinase (*Arabidopsis thaliana*)	Biological process: hormone mediated signaling pathway; protein autophosphorylation; Molecular function: ATP binding; peptide receptor activity; transmembrane receptor protein serine/threonine kinase signaling pathway; ubiquitin protein ligase binding Cellular component: cell wall; integral component of membrane; plant-type cell wall; plasma membrane; plasmodesma
c52051_g2^∗^	LAC15^2^	0.019	–7.47	Laccase-15 (*Oryza sativa* subsp. *japonica*)	Biological process: lignin catabolism Molecular function: copper ion binding, hydroquinone:oxygen oxidoreductase activity Cellular component: apoplast
c56238_g3	–	0.047	–9.50	B2 protein-like (*Setaria italica*)	–
c5429_g1	LECH^1^	0.004	–12.55	Horcolin (*Hordeum vulgare*)	Molecular function: mannose binding Cellular component: apoplast

*Annotation and/or product descriptions are based on Blast searches (*E*-value ≤ 0.05). Gene Ontology (GO) terms as given in UniProtKB where available. Sequences marked ^∗^ are those unique to the ‘L-16 cf. H-16’ treatment comparison shown in **Figure 3**. ^1^Blastp Swiss-Prot; ^2^Blastx Swiss-Prot; ^3^Blastx nr GenBank; ^4^Blastn nt GenBank.*

Despite initial expectations that *P. australis* from various sites in the Gippsland Lakes would not grow at salinities >10 g L^-1^ (based on field data presented in Bird, 1961), specimens from all six sites grew well (albeit often with reduced vigor) at the highest salinity used in the trial, 16 g L^-1^ (Boon et al., 2015c). Specimens collected from the most saline site - Cunninghame Arm - were unaffected by the highest salinity used in the growth trials. At 8 g L^-1^ and 16 g L^-^^1^ their final above-ground biomass was indistinguishable from plants grown at 0 g L^-1^. Plants collected from Ocean Grange, another highly saline site, were unaffected by salinity over the range 0-8 g L^-1^, but showed a 59% reduction in biomass at 16 g L^-1^. Similar responses were observed when plant performance was gauged in terms of plant height. When *P. australis* was grown in freshwater conditions plants ranged in height from ∼0.6–1.2 m at the end of the growth trials. Mean plant height was little affected by salinity up to 4 g L^-1^, and at higher salinities effects were dependent upon the plants’ provenance. The height of specimens from Ocean Grange, for example, was little affected at 8 g L^-1^ whereas heights of plants from the three low salinity river sites – Avon, Tambo and Nicholson – were reduced by ∼50–60% relative to the 0 g L^-1^ controls. At the highest salinity, mean plant heights were reduced by ∼60–70%, except for specimens collected from Ocean Grange (35%).

## Discussion

### General Response of *Phragmites* to Salinity

In this study, we present the first comparative transcriptome data for culms of *Phragmites australis* and the first transcript data from southern hemisphere clones of this species. We found that a broad suite of genes was significantly up- or down-regulated in *P. australis* culms in response to irrigation with freshwater, relative to saline water. Changed levels of expression were found in *P. australis* in genes including those identified in other plant species as being responsive to osmotic stress such as DHN1 and DHN3 (coding for dehydrin proteins) (Singh et al., 2015).

However, in our study the expression levels of many of the genes commonly associated with a response to salt stress in plants were not significantly different in *P. australis* clones irrigated with saline compared to fresh water (**Table 3**). These genes included those previously identified as varying between salt tolerant and salt sensitive plants of *P. australis* (Zhao et al., 2004; Takahashi et al., 2007a,b,c, 2009; Eller et al., 2014) and in other species (e.g., Munns and Tester, 2008) including the HAK/HKT gene family. Several genes that have previously been linked to salt stress in other plant species were significantly down-regulated, or the expression did not differ between *P. australis* plants exposed to saline water compared to fresh water while we had predicted significant up-regulation. Some of the discrepancy between our results and those of other researchers might be explained by our comparison of genotypes that are more closely related than those compared in other studies.

**Table 3:** Examples of genes associated with salt stress response in plants and their relative expression levels in culms of *Phragmites australis* for the ‘L-0 cf. L-16’ comparison in this study.

Gene	Product	Organism	Contig sequence ID	Log_2_-fold change	FDR
***CIPK24***	CBL-interacting serine/threonine-protein kinase 24 (SOS2)	*Oryza sativa* subsp. *indica*	c58974_g1	–1.81	0.66
***CNBL4***	Calcineurin B-like protein 4 (SOS3)	*O. sativa* subsp. *indica*	c43983_g1	–0.51	0.94
***FLA4***	Fasciclin-like arabinogalactan protein 4 (SOS5)	*Arabidopsis thaliana*	c67150_g1	1.18	0.9
***GPX4***	Glutathione peroxidase	*A. thaliana*	c51621_g4	–0.79	0.73
***HAK26***	Potassium transporter 26	*O. sativa* subsp. *japonica*	c36769_g1	–3.35	0.33
***HKT7***	Probable cation transporter	*O. sativa* subsp.*japonica*	c73767_g1	–3.63	0.62
***HSP26***	26.7 kDa heat-shock protein	*O. sativa* subsp. *japonica*	c49650_g1	4.53	0.02
***MSD2***	Superoxide dismutase [Mn] 2, mitochondrial	*A. thaliana*	Not found	-	-
***MYB4***	Myb-related protein	*O. sativa* subsp. *japonica*	c51737_g4	–4.78	0.25
***NHX7***	Sodium/hydrogen exchanger 7 (SOS1)	*A. thaliana*	c34293_g2	0.44	0.9
***PERK2***	Proline-rich receptor-like protein kinase	*A. thaliana*	c46152_g3	–1.45	0.64
***PGKH***	Phosphoglycerate kinase, chloroplastic	*Triticum aestivum*	c36562_g1	–0.51	0.93
***PhaNHA1***	Na^+^/H^+^ antiporter	*Phragmites australis*	Not found	-	-
***PK***	Pyridoxal kinase (SOS4)	*A. thaliana*	c32545_g1	–1.09	0.72
***SALT***	Salt-stress-induced protein	*O. sativa* subsp. *indica*	c72546_g1	2.89	0.72

*Where a gene is part of a family (e.g., HAK), the copy with the lowest False Discovery Rate (FDR) and most extreme log_2_-fold change is listed. FDR ≤ 0.05 is considered significant.*

This was the case for clones from both low- and high-salinity populations within the Gippsland Lakes. A possible explanation for this discrepancy is that these genes are more strongly expressed in tissues other than culms (e.g., roots and rhizomes) when the *P. australis* clones are exposed to salt stress. In addition, there are likely to be changes in the suite of genes expressed, or their expression levels, related to the period of exposure to salt stress and the stage of physiological response (see Munns and Tester, 2008). Another possible source of variation is that surface-water measurements did not always represent the salinity to which plants were exposed. This can be largely discounted because, as noted earlier, there was a good correlation between surface- and interstitial-water salinities at the various field-collection sites. This indicates that plants growing in saline sites were unlikely to be subsidized by a shallow lens of fresh ground water. It remains possible that some interconnections persist via rhizomes between widely spaced stems in large clones and that sections of a genet growing in lower salinity could alleviate stressors in those sections exposed to higher salinity. However, this scenario seems unlikely due to the regular and consistent salinity regimes that existed within a given site. While this varied intra-genet salinity exposure may occur where a clone expands across a strong salinity gradient, it seemed not to be the case in any of the sites from which plants were collected for this study.

### Differential Response of *Phragmites* Clones to Salinity

We found clear differences in gene expression responses to salinity treatment between *P. australis* clones sourced from low salinity areas compared to those sourced from highly saline sites within the Gippsland Lakes. This suggests *in situ* local adaptation of clones within this species to varied salinity levels.

When irrigated with highly saline water, *P. australis* clones with a highly saline provenance (BB, CA, and OG) displayed considerably higher expression levels for genes coding for Dirigent 1 protein (DIR1) and Chlorophyll a/b-binding protein 48 (CB48), than clones from low salinity sites (A, N, TR). DIR proteins are hypothesized to play a role in lignan biosynthesis in the presence of laccase (Davin et al., 1997) and Chlorophyll a/b binding protein 48 is part of the antenna system of the photosynthetic apparatus (Knight et al., 1992; Jansson, 1994). This result suggests that plants from highly saline sites in the Gippsland Lakes are able to maintain higher levels of photosynthesis and biomass production than those from low salinity sites under such conditions, and is supported by growth data for these plants collected in a parallel study (Boon et al., 2015c). Of note, a field-based study of *P. australis* in the Gippsland Lakes indicated reduced photosynthetic efficiency of plants from high salinity sites relative to those in low salinity sites as measured by leaf fluorescence (*Fv*/*Fm*) (Hurry et al., 2013). Their data indicated that plants in highly saline conditions were exhibiting at least some degree of stress response regardless of provenance; a finding paralleled in a greenhouse-based study by Achenbach et al. (2013).

Achenbach et al. (2013) also found differential growth/photosynthesis response to salinity in *P. australis* clones sourced from a range of ploidy levels and geographic areas. However, these authors were looking at global scale differences with plant material sourced thousands of kilometers apart. In the current study, such differences were observed for plants of a (putative) single ploidy level at a scale of tens of kilometers in a single lake system.

While we observed different responses to salt exposure, Hurry et al. (2013) found only moderate genetic structure based on neutral genetic markers (microsatellites) in *P. australis* across the Gippsland Lakes and no correlation between genetic structure and water salinity. In contrast, Gao et al. (2012) found genetic structure based on microsatellite markers in *P. australis* in the Yellow River Delta associated with soil salinity and over a similar geographic scale. The cause of this discrepancy is unclear, but may be related to the suite of markers used, the degree of gene-flow or clonal spread amongst sites, or factors unaccounted for such as temporal changes in salinity. This highlights the need to identify genetic responses affecting biological functions that are likely to be responsive to directional selection.

As the Gippsland Lakes complex is over 120 km long and is fed by seven major inflowing freshwater rivers (**Figure 1**), there exists a wide range of soil/water salinity conditions to which *P. australis* can be exposed and which may vary temporally. When rhizomes were collected for our study in spring 2014, surface water salinity across the system ranged from 1.2 to 36 g L^-1^ TDS and was positively correlated with the salinity of subsurface soils (Boon et al., 2015c). The degree and frequency of changes to salinity levels at these sites is uncertain but will have an impact on the growth of clones and favor those whose response can minimize the detrimental effects on growth.

When plants are grown in highly saline water, a transcript showing a close affinity with the gene coding for Horcolin (*Hordeum vulgare* coleoptile lectin) was strongly up-regulated in clones from two of the three low salinity sites (A and N) relative to clones from high salinity sites but down-regulated slightly in the clones from the third low-salinity site (TR). This protein is hypothesized to be a mannose binding lectin (Grunwald et al., 2007) and may play a role in the context of stress signaling in plants (Yang et al., 2013). The significance of this putative stress response in *P. australis* is unclear, particularly given the varied response among clones from low salinity sites. Variation in response at the clonal level as well a treatment level highlights the difficulty in identifying the underlying genetic mechanisms of salinity response under field conditions.

The transcriptome data presented here, coupled with the field-based experimental design, provides a deeper understanding of the complex responses to salt stress in *P. australis*. Our results have shown that a transcriptomics approach provides useful new data, although there are limitations to what can be inferred. We have only examined variation in gene expression levels in *P. australis* culms whereas much of the primary physiological response to salt stress may be specific to tissues within the plant roots and rhizomes. Response may also vary temporally (see Munns and Tester, 2008) so a comparison of responses in different tissues and at different times after exposure to salinity is needed to provide a clearer understanding of differences in salt tolerance among *P. australis* clones.

While we found strong evidence of differential gene expression in culms among *P. australis* clones and salinity treatments, the underlying reasons for this variation are likely to be complex and involve many gene-by-environment interactions. In addition, the variable response of clones to salinity may be influenced by other factors including endophytic or mycorrhizal relationships. The beneficial role of symbionts in imparting increased salinity tolerance for grasses has been demonstrated by several researchers (e.g., Feng et al., 2002; Saleh Al-Garni, 2006; Baltruschat et al., 2008). Supporting these findings, Ma et al. (2013) showed differences in bacterial and archaeal endophyte assemblages in the tissues of *P. australis* growing along a salinity gradient and Kowalski et al. (2015) have recently investigated the potential for indirectly controlling invasive *P. australis* growing around the Great Lakes in North America by manipulating the occurrence of beneficial microfungal endophytes. Epigenetic variation may also influence variation in salt tolerance among, and within *P. australis* clones in the absence of underlying sequence variation (e.g., Richards et al., 2012; Foust et al., 2016). Epigenomic analysis of different clones could be used in conjunction with transcriptomic data to determine whether non-sequence-based differences influence salinity tolerance and are heritable.

Our study is one step toward developing a series of genetic markers that can be used to select genetically diverse, salt tolerant germplasm for restoration purposes where salinity has been identified as a risk factor. Revegetation projects involving *P. australis* could be more effective if they utilize multiple salt-tolerant clones so that high levels of genetic diversity are maintained to enable adaptive responses. Plant response to environmental change is complex and further study is required to understand how genetic and environmental factors interact to influence salinity tolerance and adaptive capacity in *P. australis*.

While we have focussed on restoration of *P. australis* in salinizing wetlands, the ability to identify genes that confer salinity tolerance also has implications for understanding why particular lineages may be more competitive under saline conditions. We have seen differences in the relative levels of gene expression and this approach provides a means to investigate lineages that are declining or becoming invasive. For example, different gene expression levels may provide insight into the invasiveness in North America of the introduced Eurasian lineage (haplotype M, *sensu* Saltonstall, 2002, 2003) which has a faster growth rate under saline conditions compared to the native North American subspecies (Vasquez et al., 2005, 2006; Howard et al., 2007). At the same time, the approach of using salt tolerant germplasm for restoration must be applied with caution, as there is still a risk that specific native clones and/or lineages could become invasive and dominate sites, particularly if few clones are used widely for restoration.

In our study, clonal differences in gene expression suggest that those from highly saline areas in the Gippsland Lakes are better able to maintain effective physiological functions under saline conditions relative than those from freshwater areas. This has implications for biological conservation and restoration of *P. australis* in temperate coastal wetlands worldwide where increasing salinization is a consequence of environmental changes.

## Availability of Supporting Data

The transcriptome sequence dataset supporting the results in this article is available from the NCBI Sequence Read Archive, accession SRR3233385-SRR3233398. The GenBank BioProject Accession number is PRJNA314710. This Transcriptome Shotgun Assembly project has been deposited at DDBJ/EMBL/GenBank under the accession GEKX00000000. The version described in this paper is the first version, GEKX01000000.

## Author Contributions

PB and EJ conceived the broad experiment and were the recipients of GLEF project funds; EJ conceived the molecular analysis. GH, EJ, and PB collected samples. PB prepared and supervised the growth trial. AG provided laboratory space, equipment, and reagents. GH undertook cDNA library preparation and data analysis. NH facilitated sequencing of the cDNA libraries and undertook bioinformatics. GH, EJ, and PB prepared the manuscript.

## Conflict of Interest Statement

The authors declare that the research was conducted in the absence of any commercial or financial relationships that could be construed as a potential conflict of interest.

## Footnotes

1 https://nematodegenetics.wordpress.com/protocols/

2 http://trinotate.github.io

## Supplementary Material

DATA SHEET 1 **List of putative genes (contigs) with significantly different relative expression levels (FDR cut-off ≤ 0.05) identified in *Phragmites australis* clones sourced from sites in the Gippsland Lakes with high (H) or low (L) water salinity and irrigated *ex situ* with either fresh (0) or 16 g L^-1^ TDS saline water (16).** Each worksheet lists different pair-wise comparisons of assembled transcript data. Relative expression levels are given as log_2_-fold change and read numbers for the individual samples listed as counts per million reads. Population codes are as listed in **Table 1**.

DATA SHEET 2 **Lists of putative genes (contigs) with significantly different relative expression levels (FDR; α ≤ 0.05) that were common to multiple pair-wise treatment regimes in *Phragmites australis* clone culms.** These details correspond to the results displayed in Venn diagrams presented in **Figure 4** of the main text. Data in the Blastp and Blastx columns represent significant Blast hits against the Swiss-Prot/UniProtKB databases and display UniProt annotations while Blastx nr and Blastn nt represent significant (*E*-value ≤ 0.05) hits against GenBank.
